# Comparative pathologic analysis of mediastinal B-cell lymphomas: selective expression of p63 but no GATA3 optimally differentiates primary mediastinal large B-cell lymphoma from classic Hodgkin lymphoma

**DOI:** 10.1186/s13000-019-0918-x

**Published:** 2019-12-12

**Authors:** Hyun-Jung Kim, Hee Kyung Kim, Gyeongsin Park, Soo Kee Min, Hee Jeong Cha, Hyekyung Lee, Suk Jin Choi, Hee Young Na, Ji-Young Choe, Ji Eun Kim

**Affiliations:** 1 Departments of Pathology, Inje Univ. Sanggye Paik Hospital, Seoul, South Korea; 2 Departments of Pathology, Soonchunhyang Univ. Bucheon Soonchunhyang Hospital, Bucheon, South Korea; 3 Departments of Pathology, Seoul St. Mary’s Hospital, Catholic University of Korea, Seoul, South Korea; 4 Departments of Pathology, Hallym University Sacred Heart Hospital, Anyang, South Korea; 5 Departments of Pathology, Ulsan University Hospital, Ulsan, South Korea; 6 Departments of Pathology, Eulji University Hospital, Daejon, South Korea; 7 Departments of Pathology, Inha University Hospital, Incheon, South Korea; 8 Departments of Pathology, Seoul National University Bundang Hospital, Seongnam, South Korea; 9 Department of Pathology, SMG-SNU Boramae Medical Center, Seoul National University College of Medicine, Seoul, South Korea

**Keywords:** Primary mediastinal large B-cell lymphoma, Classic Hodgkin lymphoma, p63, GATA3, Cyclin E, Immunohistochemistry

## Abstract

**Background:**

Interpretation of mediastinal biopsy is often challenging even for experienced pathologists especially when a hematolymphoid neoplasm is suspected. Primary mediastinal large B-cell lymphoma (PMLBCL) and classic Hodgkin lymphoma (CHL) represent two major types of mature B-cell lymphomas of the mediastinum. Although PMLBCL and mediastinal CHL share many clinicopathologic characteristics, their treatment strategies and responses are remarkably different. We therefore aimed to find distinctive histologic or protein markers to better differentiate these two lesions.

**Methods:**

Search for primary mediastinal B-cell lymphomas found 52 consecutive cases from 3 university hospitals of Korea during 2005 to 2012. Among them, 32 cases that were available for additional immunohistochemistry (IHC) assessment were enrolled in this study. These cases consisted of the following: CHL (*N* = 13), PMLBCL (*N* = 16), and B-cell lymphoma unclassifiable, with features intermediate between diffuse large B-cell lymphoma and CHL (gray zone lymphoma, *N* = 3). Along with the clinicopathologic findings, the expression of p63, GATA3 and cyclin E was investigated by IHC in the three categorized lesions mentioned above.

**Results:**

Most clinical features overlapped between PMLBCL and CHL except for the increased disease progression and mortality found in PMLBCL. In the pathologic review, the presence of epithelioid granuloma favored a diagnosis of CHL, whereas reticulated or alveolar patterns of fibrosis were characteristic of PMLBCL. For protein markers, p63 was predominantly positive in PMLBCL (15/16) compared with CHL (2/13), which indicates that p63 is a marker of the highest diagnostic accuracy when calculated by the area under the ROC curve. GATA3 was expressed in the majority of CHL cases (10/13) compared with PMLBCL (0/16), while the expression of cyclin E was only rarely present in a minor population of PMLBCL.

**Conclusions:**

P63 expression in tumor cells, even focal expression, and no GATA3 is the most helpful feature in distinguishing PMLBCL from mediastinal CHL.

## Background

Malignant lymphoma is one of the most common causes of bulky anterior mediastinal masses in young patients [1]. Lymphomas of the mediastinum can originate either from the thymus or the lymph nodes, and thymic lymphomas show unique pathologic characteristics distinct from common nodal lymphomas [2]. Two primary representative T- and B-cell lymphomas arise in the thymus: precursor T-lymphoblastic lymphoma/leukemia (T-LBL) and primary mediastinal large B-cell lymphoma (PMLBCL). In T-LBL, prompt pathologic diagnosis is critical because the tumor cells are highly proliferative and frequently give rise to acute airway compression. However, T-LBL can be relatively easily excluded because of its uniform histologic features and by confirmation of the pathognomonic marker TdT by immunohistochemistry (IHC). On the contrary, the differential diagnosis of B-cell lymphomas is much more complicated. PMLBCL and classic Hodgkin lymphoma (CHL) constitute two major lymphomas of mediastinal B-cell origin, although other types of B-cell lymphomas such as marginal zone lymphoma do occur at a low incidence. PMLBCL and CHL share a considerable degree of similarity in terms of histomorphology, immunoprofiles and even gene expression patterns [3, 4]. The intrinsic nature of thymic B-cells and their microenvironment are responsible for the clinicopathologic features of these two lesions. Nevertheless, differentiation of PMLBCL from CHL or other B-cell lymphomas is crucial even in small specimens, because they each have different therapeutic strategies [5] and outcomes. However, histomorphologic features are frequently obscured by crush artifacts, which are almost inevitable in mediastinal biopsy due to limitations of the surgical approach. Therefore, recognition of the salient histologic patterns and careful selection of protein markers to minimize tissue loss should be pivotal in the pathologic diagnosis. This study aimed to find histopathologic parameters and immunohistochemical markers that will best characterize PMLBCL and CHL.

## Methods

### Case selection

Cases of B-cell lymphomas arising in the mediastinum from 2005 to 2012 were retrospectively reviewed from the archives of three university hospitals in Korea. Cases were selected when patients had solely mediastinal masses without lymphadenopathy of the other sites or when patients had a predominantly mediastinal presentation if accompanied by other nodal lesions. Histopathologic and immunohistochemical findings were reviewed at a consensus meeting of five board-certified pathologists (HJK, JEK, GSP, HKK, SKM) who agreed to use the diagnostic criteria of the 2017 WHO classification tumours of haematopoietic and lymphoid tissues [6]. Patients’ demographic data and other clinical characteristics along with follow-up data were retrieved from the electronic medical records.

### Expression of protein markers and detection of EBV

Representative tissue sections that were 4 μm in thickness were used for IHC and EBV-encoded RNA (EBER) in situ hybridization (ISH). The primary antibodies used for routine pathologic examination are: CD3, CD5, CD10, CD15, CD20, CD23, CD30, CD79a, BCL6, Pax5, and IRF4/MUM-1. Additionally, IHC for GATA3 (Biocare Medical, Concord, CA), p63 (Lab Vision, Fremont, CA) and cyclin E (Santacruz Biotechnology, Santa Cruz, CA) was performed to determine their usefulness in the differential diagnosis. Expression of p63, GATA3 and cyclin E was considered positive if the percentage of positive cells was equal to or higher than 5%. To elucidate whether those three proteins are site specific marker, 88 cases of non-mediastinal DLBCL and 56 non-mediastinal CHL were investigated using tissue microarray blocks. All IHC and EBER ISH assays were conducted using the Bench Mark automated immunostainer (Ventana Medical Systems, Tucson AZ) according to validated protocols.

### Statistical analysis

Nonparametric Mann-Whitney U-test and Fisher’s Exact Test were performed to compare IHC and EBER ISH results between each group of mediastinal B-cell lymphoma. Spearman’s rho was used to assess the correlation between protein expression scores. Overall survival (OS) was calculated from the date of diagnosis to the date of death and was compared using univariable Kaplan-Meier survival functions with log rank tests. The area under the receiver operating characteristics curve tested by the DeLong test was applied to determine the most discriminative diagnostic marker [7]. The results were considered statistically significant if a two-tailed *P*-value was < 0.05. All data were analyzed with SPSS software version 20.0 (SPSS Inc. IBM, NY, USA).

## Results

### Patient profile

**Fig. 1:**
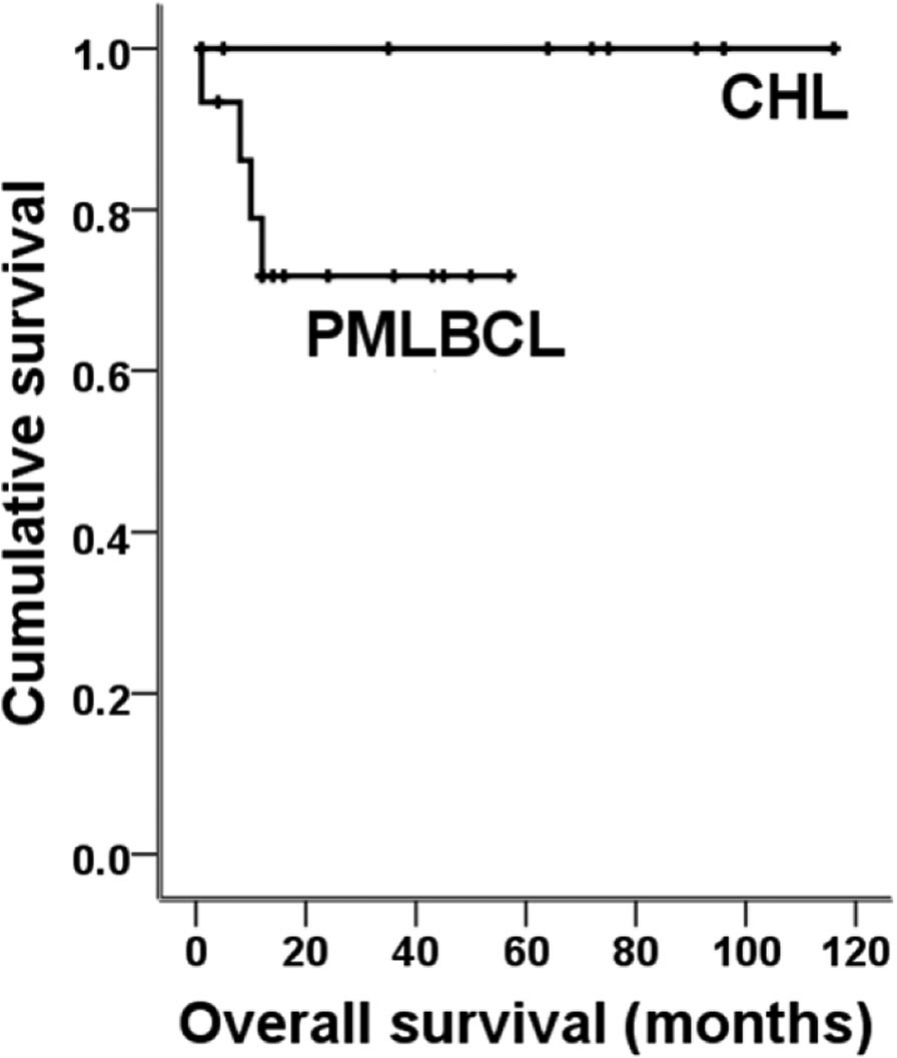
Overall survival of patients with primary mediastinal large B-cell lymphoma and classic Hodgkin lymphoma

Initially, 52 cases of mediastinal lymphomas of B-cell origin were reviewed and biopsy materials were obtained by transthoracic needle biopsy (*N* = 32), video-assisted thoracoscopic surgery (*N* = 12) or open surgical biopsy (*N* = 8). Half of the needle biopsy specimens (16 of 32) were removed from the study largely due to shortfall of samples either by quality- or quantity-based standards, and 4 cases of other type of B-cell lymphomas were also excluded because they were outside the scope of the study. Therefore, 32 cases were finally selected for this study: PMLBCL (*N* = 16), CHL (*N* = 13), B-cell lymphoma, unclassifiable, with features intermediate between DLBCL and classical Hodgkin lymphoma (gray-zone lymphoma) (*N* = 3). The detailed demographic data and survival plot of the patients are shown in Table 1 and Fig. 1. Cases were predominantly female, and the patients’ ages ranged from 15 to 86 years. Most PMLBCL patients were treated with rituximab-based chemotherapy such as R-CHOP (rituximab, cyclophosphamide, doxorubicin, vincristine, and prednisone) (13 of 16, 81.3%), while most CHL patients (12 of 13, 92.3%) were managed by ABVD (doxorubicin, bleomycin, vinblastine, and dacarbazine). Three patients with gray zone lymphoma were treated with the R-CHOP regimen because all had been previously diagnosed with DLBCL; however, the diagnosis was revised to gray zone lymphoma after review at the consensus meeting. The median follow-up was 72 months (range 1–116 months) for CHL, and 13 months (range 1–57 months) for PMLBCL. Cancer-specific death occurred in 5 patients, four of whom were diagnosed with PMLBCL and one of whom was diagnosed with gray zone lymphoma. Compared with patients with CHL, patients with PMLBCL showed significantly higher incidences of disease progression (*p* = 0.017) and a tendency for shorter OS (*p* = 0.08) (Fig. 1).

**Table 1:** Clinical profile of patients with B-cell lymphoma of the mediastinum

		CHL, NS (*N* = 13)	PMLBCL (*N* = 16)	*p* value	Gray zone (*N* = 3)
Age	Median (Range)	26(15–73)	30 (19–51)	0.175	28 (25–52)
Sex	Male: Female	4(30.8%): 9(69.2%)	6(37.5%): 10(62.5%)	1.000	2: 1
Methods of biopsy	Needle: VATS: Open	6: 3: 4	10: 4: 2		0: 0: 3
IPI score	High risk (> 3) or low	7(87.5%): 1(12.5%)	13(86.7%): 2(13.3%)	1.000	1: 2
Ann Arbor stage	I-II: III-IV	8(80.0%): 2(20.0%)	10(71.4%): 4(28.5%)	1.000	1: 2
LDH	Elevated or not	4(57.1%): 3(42.9%)	12(80.0%): 3(20.0%)	0.334	0: 3
ECOG score	≥2 or less	0: 12(100%)	0: 14(100%)		0: 3
Bone marrow	Involved or not	1(9.1%): 10(90.9%)	2(13.3%): 13(86.7%)	1.000	0: 3
Bulky disease	Yes or No	6(54.5%): 5(45.5%)	11(73.3%): 4(26.7%)	0.419	1: 2
B symptoms	Yes or No	5(55.6%): 4(44.4%)	4(28.6%): 10(71.4%)	0.383	1: 2
Follow-up (months)	Median (Range)	72 (1–116)	14(1–57)		48 (14–84)
	Dead/Alive	0: 12(100%)	4(26.7%): 11(73.3%)	0.106	1: 2
Progression	Yes or No	0: 12(100%)	6(42.9%): 8(57.1%)	0.017	1: 2

*CHL* classic Hodgkin lymphoma, *NS* nodular sclerosis, *PMLBCL* primary mediastinal diffuse large B-cell lymphoma (DLBCL); *Gray, B-cell lymphoma* unclassifiable, with features intermediate between DLBCL and classic Hodgkin lymphoma; *VATS* video-assisted thoracoscopic surgery

### Comparison of the pathologic features between PMLBCL and CHL

**Fig. 2:**
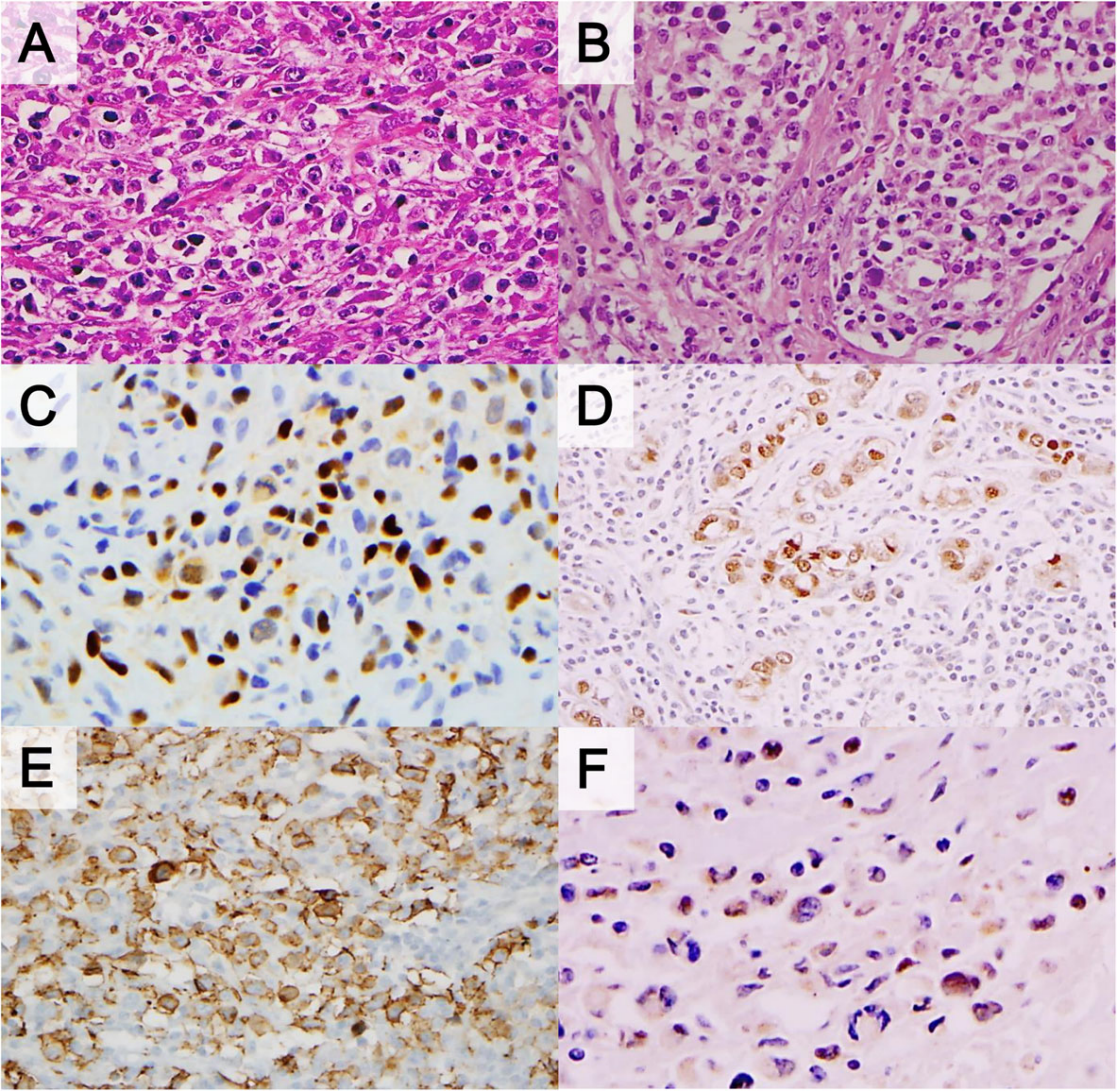
Pathologic findings of primary mediastinal large B-cell lymphoma. Reticular fibrosis (**a**) or alveolar type tumor aggregates (**b**) is characteristic. Expression of p63 (**c**), bcl-6 (**d**), and CD23 (**e**) were significantly higher than that of classic Hodgkin lymphoma. Minority of cases showed focal positivity for cyclin E (F)

**Fig. 3:**
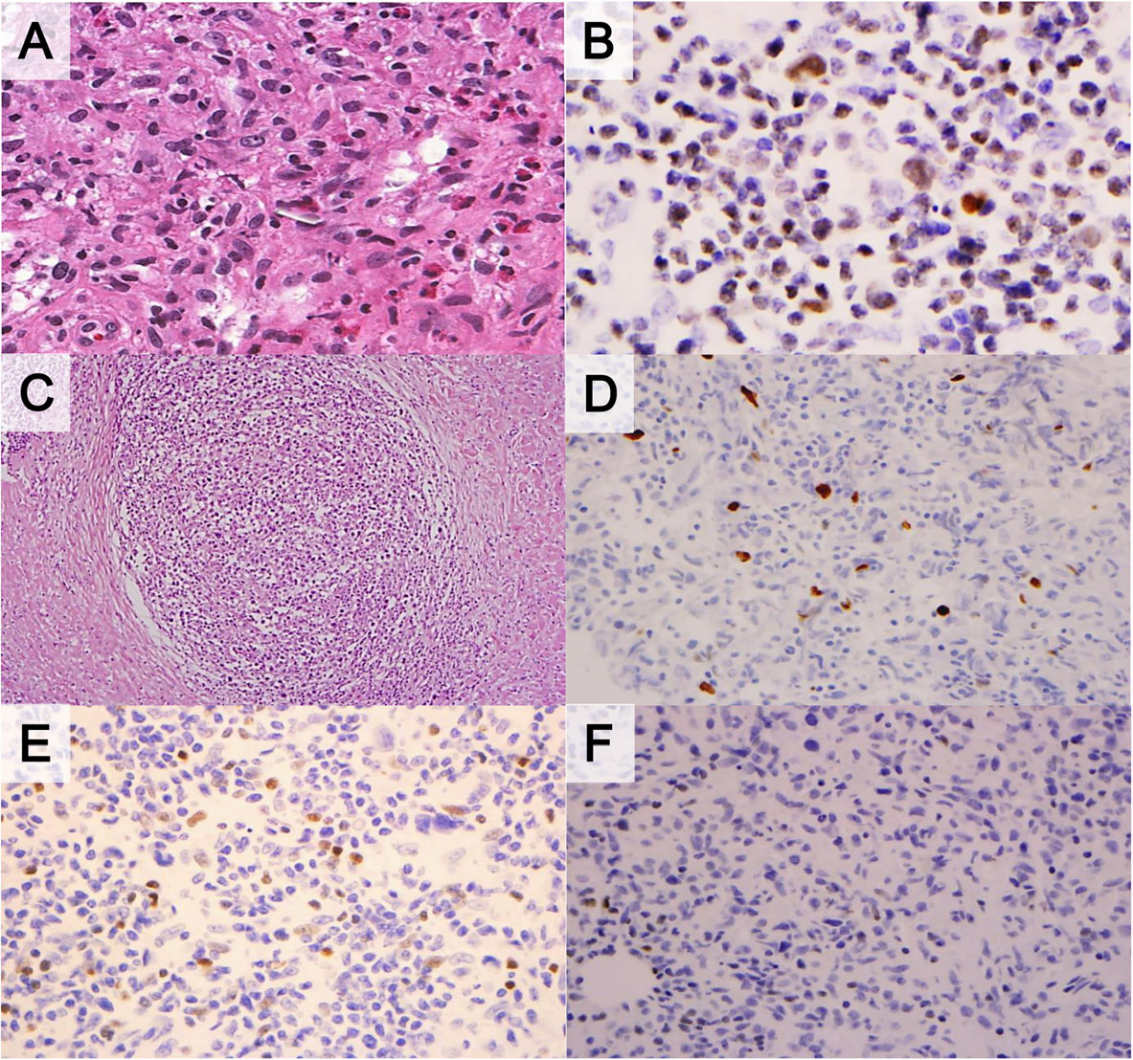
Classic Hodgkin lymphoma (**a**) showed significantly higher positivity for GATA3 (**b**). A case of gray zone lymphoma showed nodular fibrosis (**c**), focal positivity for MUM1 (**d**), p63 (**e**) and negativity for GATA3 (**f**)

The review of the pathological findings (Table 2, Figs. 2 and 3) revealed significant differences between PMLBCL and CHL with respect to the presence of compartmentalizing alveolar pattern fibrosis (0 vs. 75%), dense collagenous bands (0 vs. 53%), diffuse sheet-like expression of CD20 (0 vs 62.5%) and CD30 (100% vs 37.5%), and positivity for CD23 (7.7% vs 37.5%), bcl-6 (0 vs 56.2%) and IRF4/MUM1 (100% vs 50%), p63 (15.4% vs 93.8%) and GATA3 (76.9% vs 0). Cyclin E was completely negative in all CHL cases, whereas a low frequency (two of 16 PMLBCL cases) of positive tumor cells was observed. An inflammatory background including granuloma or nodularity was not infrequently shared in the two disease groups. Among these pathologic parameters, expression of p63 or GATA3 and the presence of alveolar fibrosis were found to be extremely sensitive and specific markers for the differentiation of CHL from MLBCL after calculating the area under the receiver operating characteristics (Table 3). Additionally, expression of p63 and GATA3 were compared with non-mediastinal CHL and DLBCL (Additional file 1: Table S1 and Additional file 2: Table S2). GATA3 was preferentially present in tumor cells of mediastinal CHL rather than non-mediastinal origin (*p* = 0.000).

**Table 2:** Comparison of pathologic parameters

		CHL, NS (*N* = 13)	PMLBCL (*N* = 16)	*p* value	Gray (*N* = 3)
Granuloma	Present or absent	3(23.1%):10(76.9%)	0:16(100%)	0.078	0:3
Fibrosis	Diffuse/nodular/reticular/none	8(61.5%):5(38.5%):0:0	1(6.2%):1(6.2%):12(75.0%):2(12.5%)	< 0.001	1:2
CD20	Negative/focal/diffuse	11(84.6%):2(13.4%):0	0:6(37.5%):10(62.5%)	< 0.001	0:3:0
CD23	Positive (> 30%) or negative	1(8.3%):11(91.7%)^a^	6(37.5%):10(62.5%)	0.024	2:1
CD30	Diffuse/ focal/negative	13(100%):0:0	6(37.5%):6(37.5%):4(25.0%)	0.001	2:1
IRF4/MUM1	Positive (> 30%) or negative	13(100%):0(0%)	8(50.0%):8(50.0%)	0.003	2:1
BCL6	Positive (> 30%) or negative	0:12(100%)^a^	9(56.2%):7(43.8%)	0.003	1:2
CD10	Positive (> 30%) or negative	0:12(100%)^a^	1(6.3%):15(93.7%)	1.000	0:3
COO	GC or non-GCB	0:12(100%)^a^	6(37.5%):10(62.5%)	0.024	0:3
EBV	Present or absent	1(7.7%):12(92.3%)	1(6.2%):15(93.8%)	1.000	0:3
P63	Positive (> 5%) or negative	2(15.4%):11(84.6%)	15(93.8%):1(6.2%)	< 0.001	2:1
GATA3	Positive (> 5%) or negative	10(76.9%):3(23.1%)	0:16(100%)	< 0.001	2:1
CyclinE	Positive (> 5%) or negative	0:13(100%)	2(12.5%):14(87.5%)	0.492	0:3

*CHL* classic Hodgkin lymphoma, *NS* nodular sclerosis, *PMLBCL* primary mediastinal diffuse large B-cell lymphoma (DLBCL); *Gray, B-cell lymphoma* unclassifiable, with features intermediate between DLBCL and classic Hodgkin lymphoma

^a^: immunohistochemistry for some antibodies was not done in one case due to exhaustion of stored tissues

**Table 3:** Diagnostic utility of pathologic markers in mediastinal B-cell lymphomas

	Specificity (CHL, NS)	Sensitivity (PMLBCL)	NPV (CHL, NS)	PPV (PMLBCL)	AUC (95% CI)	p value	DeLong test (*p* value)
P63	84.6%	93.8%	91.7%	88.2%	0.892 (0.756, 1.000)	< 0.001	reference
GATA3	75.0%	100.0%	100.0%	84.2%	0.875 (0.721, 1.000)	0.001	0.918
IRF4/MUM1	100.0%	50.0%	61.9%	100.0%	0.750 (0.570, 0.930)	0.023	0.133
BCL6	100.0%	56.2%	63.2%	100.0%	0.781 (0.609, 0.954)	0.012	0.276
CD30	100.0%	62.5%	68.4%	100.0%	0.813 (0.651, 0.974)	0.004	0.386
CD23	87.5%	71.4%	63.6%	90.9%	0.795 (0.595, 0.994)	0.024	0.745
EBV	92.3%	6.2%	44.4%	50.0%	0.493 (0.278, 0.708)	0.948	
CyclinE	100.0%	12.5%	46.2%	100.0%	0.563 (0.348, 0.777)	0.577	
Granuloma	23.1%	100.0%	100.0%	61.5%	0.615 (0.402, 0.828)	0.293	
Alveolar fibrosis	100.0%	75.0%	76.5%	100.0%	0.875 (0.739, 1.000)	0.001	0.844

*CHL* classic Hodgkin lymphoma; *NS*, nodular sclerosis; *PMLBCL*; primary mediastinal diffuse large B-cell lymphoma; *NPV* negative predictive value; *PPV* positive predictive value; *AUC* area under the receiver operating characteristics

We performed the DeLong test to determine whether the addition of several important pathologic variables listed above could increase the diagnostic power for CHL and PMLBCL, but p63 was demonstrated to be the single most efficient marker of PMLBCL and was superior to various combinations (Table 3).

## Discussion

In this study, we evaluated the diagnostic efficacy of several pathologic parameters including a few relatively uncommon markers in the two most representative mediastinal B-cell lymphomas. As a result, alveolar or compartmental fibrosis was the most specific and authentic histologic feature supporting a PMLBCL diagnosis. Among the protein markers, p63 and GATA3 were the most useful diagnostic markers; p63 was almost exclusively present whereas GATA3 was completely negative in PMLBCL. However, because of the interpretation difficulty of GATA3 based on the extremely low proportion of positive tumor cells, the application of p63 IHC is more highly recommended since it is easily recognizable even in small biopsy materials.

Mediastinal CHL and PMLBCL are supposedly of the same cellular origin, which has been supported by the presence of common genetic alterations and gene expression profiles [3, 8, 9], and more recently by immune-oncologic signatures such as PD-L1 or PD-L2 expression [10, 11]. The cellular origin of both tumors is thought to be post-germinal center B-cells lacking surface immunoglobulin,^7^ and almost all the Hodgkin/Reed-Sternberg (HRS) cells of CHL and quite a few tumor cells in PMLBCL express CD30. However, these two lymphomas exhibit a spectrum of histopathologic and immunophenotypic diversity based on the plasticity of B-cells, which should undergo physiologic transformation in the thymus. As is well known, tumor cells of PMLBCL often reveal large atypical cells reminiscent of HRS or even lacunar cells, therefore pathologists often encounter diagnostic dilemmas. Our study demonstrated that presence of fine reticulated or alveolar fibrosis strongly favored PMLBCL, however, this pattern cannot be easily recognizable in small biopsy materials. Substantial efforts have been made to find a good immunohistochemical marker of the mediastinal lymphomas, and recently, p63, cyclin E and GATA3 [12–14] have emerged as new diagnostic markers. P63 is one of the best available markers of basal or myoepithelial differentiation, but little is known of these proteins in hematolymphoid tumors. Expression of p63 has been reported in anaplastic large cell lymphoma [15], PMLBCL [16] and gray zone lymphoma [11] but is almost uniformly absent in CHL. In our series, p63 was demonstrated to be the most specific marker of PMLBCL, whereas expression of GATA3, even in a single HRS-like cell supports a diagnosis of CHL. As in previous studies [11, 16], the frequency of either p63- or GATA3-positive tumor cells in each case is variable but generally very low. This is the practical reason why p63 is preferable to GATA3 because of a relatively higher proportion of positive tumor cells.

The molecular mechanisms of p63 expression in PMLBCL and GATA3 expression in CHL have not been clearly elucidated. Rearrangement or extra copies of the TP63 gene were demonstrated in a subset of anaplastic large cell lymphoma but not in PMLBCL. As a tumor suppressor that is closely linked to the regulation of proapoptotic activity by p53 in the germinal center, the coordinative or reciprocal relationship between p63 and B-cell signaling is unknown [17]. Likewise, the theoretical background of GATA3 expression in CHL is also uncertain. GATA3 is T-cell transcription factor required for early T-cell development and its expression in B-cell lymphoma including CHL is exceptional. Experimental studies have revealed that deregulation of NFκB and Notch-1 contributes to GATA3 expression in HRS cells, which consequently leads to the activation of multiple cytokines that control microenvironment of CHL [12]. Until now, no genetic alterations of GATA3 have been reported, and its prognostic significance is not very high in CHL. However, GATA3 positivity in even a few HRS-like cells strongly supports the diagnosis of CHL in the mediastinum, where GATA3 frequency is particularly higher than other sites. Based on the heterogeneity of GATA3 expression pattern, the aberrancy of GATA3 in HRS more likely relies on microenvironmental factors rather than genetic alteration.

We did not find p63, GATA3, or cyclin E to be helpful for further characterizing gray zone lymphoma because they showed intermediate pattern between CHL and PMLBCL in our three cases. Our results are also in agreement with the previous report performed by Eberle et al. [11], which suggested the presence of common immunophenotypic features and genetic alterations that link gray zone lymphoma, CHL and PMLBCL and that places gray zone lymphoma in a still ambiguous category.

As shown in this study, thymic B-cell neoplasms are predominantly large cell types, but other types of B-cell lymphoma can occur [18–22]. Thymic B cells represent only 0.3% of total cellularity in the thymus, and the majority are mature B cells that would not undergo neoplastic transformation. However, in certain conditions such as myasthenia gravis or inflammation, migration of multipotent hematopoietic cells leads to B-cell proliferation and germinal center formation. In our series, two cases of CHL were found to have concomitant thymic cysts. The emergence of CHL in the preexisting thymic cysts has been reported. As in previous studies, our cases also support the idea that the pathogenesis of mediastinal CHL is associated with localized inflammation resulting in B-cell hyperplasia. The development of other types of B-cell lymphomas can be explained with a similar background.

## Conclusions

The distinction of mediastinal CHL and PMLBCL is critical but challenging. We suggest that the presence of alveolar fibrosis and p63 expression strongly indicates PMLBCL, whereas GATA3 positivity favors CHL even when GATA3 is expressed in a very low proportion of tumor cells.

## Supplementary information

**Additional file 1: Table S1.** Expression of p63 and GATA3 between mediastinal and non-mediastinal Hodgkin lymphoma.

**Additional file 2: Table S2.** Expression of p63 and GATA3 between mediastinal and non-mediastinal B-cell lymphoma.

## Data Availability

The datasets generated and/or analysed during the current study are not publicly available due the institutional review board restricts the use of the datasets to the current study only.

## Abbreviations

CHL: Classic Hodgkin lymphoma
DLBCL: Diffuse large B-cell lymphoma
EBER: EBV-encoded RNA
Gray zone lymphoma: B-cell lymphoma unclassifiable, with features intermediate between diffuse large B-cell lymphoma and classic Hodgkin lymphoma
IHC: Immunohistochemistry
ISH: In situ hybridization; OS: Overall survival
PMLBCL: Primary mediastinal large B-cell lymphoma
T-LBL: T-lymphoblastic lymphoma/leukemia
