# Supplementary material for: Comparative pathologic analysis of mediastinal B-cell lymphomas: selective expression of p63 but no GATA3 optimally differentiates primary mediastinal large B-cell lymphoma from classic Hodgkin lymphoma
Source: Diagn Pathol. 2019 Dec 12;14:133. doi: 10.1186/s13000-019-0918-x (PMC6909622; doi:10.1186/s13000-019-0918-x)
Supplement: Supplementary file 1 — Additional file 1: Table S1. Expression of p63 and GATA3 between mediastinal and non-mediastinal Hodgkin lymphoma. [file 13000_2019_918_MOESM1_ESM.docx]

Table S1. Expression of p63 and GATA3 between mediastinal and non-mediastinal Hodgkin lymphoma

|  |  | Mediastinal CHL (N=13) | Non-mediastinal CHL (N=13) | p value |  |
| --- | --- | --- | --- | --- | --- |
| P63 | P (>5%) vs N | 2:11 (20% and 40% positivity, each) | 0:56 (all negative) | NS |  |
| GATA3 | P (>5%) vs N | 10:3 (median 30, range 7-95%*) | 18:38 (median 15, range 5-50%*) | p=0.000 |  |

CHL, classic Hodgkin lymphoma; P, positive; N, negative; NS, not significant

*The median and range were calculated from the positive cases
