# Supplementary material for: Comparative pathologic analysis of mediastinal B-cell lymphomas: selective expression of p63 but no GATA3 optimally differentiates primary mediastinal large B-cell lymphoma from classic Hodgkin lymphoma
Source: Diagn Pathol. 2019 Dec 12;14:133. doi: 10.1186/s13000-019-0918-x (PMC6909622; doi:10.1186/s13000-019-0918-x)
Supplement: Supplementary file 2 — Additional file 2: Table S2. Expression of p63 and GATA3 between mediastinal and non-mediastinal B-cell lymphoma. [file 13000_2019_918_MOESM2_ESM.docx]

Table S2. Expression of p63 and GATA3 between mediastinal and non-mediastinal B-cell lymphoma

|  |  | PMLBCL (N=16) | Non-mediastinal DLBCL (N=88) | p value |
| --- | --- | --- | --- | --- |
| P63 | P (>5%) vs N | 15:1 (median 50, range 10-90%*) | 49:39 (median 30, range 2-100%*) | p=0.07 |
| GATA3 | P (>5%) vs N | 0:16 (all negative) | 0:88 (all negative) | NS |

PMLBCL; primary mediastinal diffuse large B-cell lymphoma (DLBCL); P, positive; N, negative; NS, not significant

*The median and range were calculated from the positive cases
